# Development and Validation Study of a Screening Questionnaire to Identify People Who Are Physically Inactive

**DOI:** 10.1002/puh2.70037

**Published:** 2025-02-27

**Authors:** Eero Kenttä, Harri Sievänen, Anastasiya Verho, Minna Paajanen, Timo Lukkarinen, Henri Vähä‐Ypyä, Jani Raitanen, Kari Tokola, Tommi Vasankari, Jari Parkkari

**Affiliations:** ^1^ Social Services Health Care and Rescue Services Division City of Helsinki Helsinki Finland; ^2^ Faculty of Sport and Health Sciences University of Jyväskylä Jyväskylä Finland; ^3^ The UKK Institute for Health Promotion Research Tampere Finland; ^4^ Department of Primary Health Care and General Practice University of Helsinki Helsinki Finland; ^5^ Culture and Leisure Division City of Helsinki Helsinki Finland; ^6^ Faculty of Social Sciences Tampere University Tampere Finland; ^7^ Faculty of Medicine and Health Technology Tampere University Tampere Finland

**Keywords:** mass screening, physical activity, questionnaire, sedentary behaviors, validation

## Abstract

**Objective:**

Insufficient physical activity (PA) is a well‐known risk factor for many non‐communicable diseases. This study aimed to develop a screening tool, the Helsinki Physical Activity Questionnaire (HPAQ), to identify physically inactive people at the population level and to help social and health care professionals promote PA among people at risk.

**Methods:**

Eighty‐five healthy adults wore a hip‐worn accelerometer for 7 days, after which they completed several PA questionnaires. These included some novel and several validated questions on PA. The reliability of individual questions to identify physically inactive people was assessed by correlation analysis. Logistic regression analysis was used to find the combination of questions that best identified physically inactive people.

**Results:**

The highest correlation of the screening questionnaire with the accelerometer was 0.46 (*p* < 0.001) for sedentary behavior (SB) and 0.42 (*p* < 0.001) for the total amount of moderate‐to‐vigorous physical activity (MVPA), respectively. The best pair of questions on total PA identified 64% of all inactive subjects (MVPA < 150 min/week) based on accelerometer data.

**Conclusions:**

The questionnaires developed for screening PA have a poor correlation with the accelerometer data. The screening questionnaires roughly describe PA level among middle‐aged participants, but if a more sensitive or specific method is needed, device‐based measurements are recommended.

## Graphical abstract

The aim of this study was to develop and validate a very short screening tool, the Helsinki Physical Activity Questionnaire (HPAQ), which could identify patients who do not meet the physical activity (PA) guidelines. When recognized they could be forwarded to different types of PA promotion.

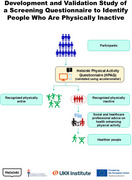

## 1 Introduction

Insufficient physical activity (PA) is a well‐known risk factor for many non‐communicable diseases [1] and premature mortality, whereas more time spent in sedentary behavior (SB) is associated with adverse health outcomes [2]. The health benefits of both regular PA and exercise are significant and undeniable [3]. Regular and diverse PA can bring numerous health benefits in the treatment of long‐term physical and mental illnesses [4, 5].

To achieve significant health benefits, adults should accumulate at least 150 min (2 h 30 min) of moderate or 75 min (1 h 15 min) of vigorous endurance‐type PA each week [6]. The recommended amount of moderate‐to‐vigorous physical activity (MVPA) can be done in bouts of a few minutes, which are known to have health benefits [7]. In addition, the PA recommendation for adults includes strength and balance exercises at least twice a week for large muscle groups [6].

When promoting a healthy lifestyle, health and social care professionals should be able to classify patients into active and inactive categories, for example, with a PA questionnaire [8]. These categories correlate with cardiovascular disease risk and reflect the importance of the dose–response relationship of PA (i.e., higher levels of PA lead to greater health benefits) [9]. The overall amount of PA can be accurately assessed with an accelerometer [10], whereas in self‐report questionnaires, participants are known to overestimate their PA and underestimate their SB [11]. According to current knowledge, questionnaires correlate poorly with PA measures in assessing PA levels [12]. However, questionnaires do have a place in situations where individuals are classified by their PA levels [2] or where physically inactive individuals are assessed for the risk of cardiovascular events during PA [13]. Other strengths of questionnaires in assessing PA are their speed of use, low cost, flexibility of data collection, ability to assess the type and context of PA, and suitability for large populations and mass screening [14]. Accelerometers, on the other hand, have limitations, such as capturing or assessing the intensity of water exercise, gym training, cycling, and rowing, and are unable to detect information about the context or type of PA [15].

The PA questionnaire should have sufficient reliability, validity, and responsiveness [16]. If these measurement properties are weak, the risk of misclassification and biased results increases [17, 18]. In validation studies of PA questionnaires, accelerometers, doubly labeled water, pedometers, or other PA questionnaires are often used as a reference method [19]. As self‐report questionnaires can overestimate the amount of PA, accelerometers are often used for validation [20].

The aim of this study was to develop a screening tool, the Helsinki Physical Activity Questionnaire (HPAQ), to identify physically inactive people and to evaluate possibilities to integrate this tool into a digital client portal for social services and health care. Unlike generally used questionnaires (i.e., the International Physical Activity Questionnaire [IPAQ] and the Global Physical Activity Questionnaire [GPAQ]), the HPAQ developed and validated in this study is not intended to assess the exact amount of MVPA or to estimate the total amount of MVPA at the population level. Instead, it aims to roughly categorize respondents into two groups (inactive and sufficiently physically active) within the social and health care context. Therefore, the developed HPAQ questionnaire contains much fewer questions than the generally used PA questionnaires, which is expected to increase its feasibility in clinical practice. The primary goal was to validate the PA screening questionnaires against accelerometer data. The secondary goal was to find a combination of questions that could best predict physical inactivity with sufficient reliability.

## 2 Methods

### 2.1 Study Population

**FIGURE 1:**
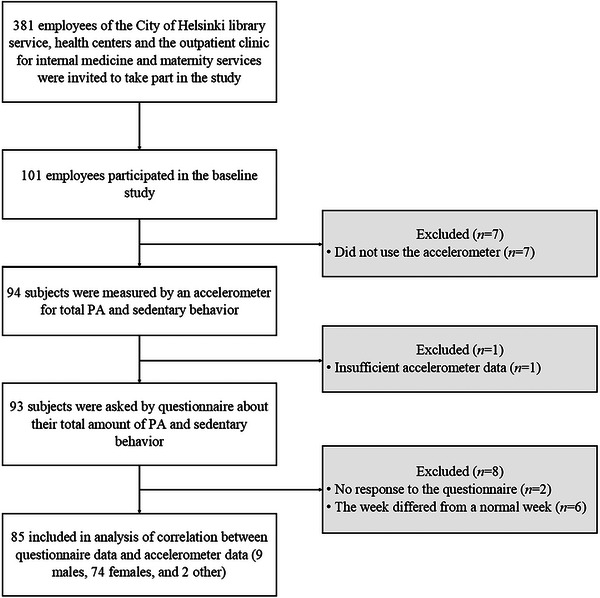
Flow chart of the study.

An invitation to the study was sent to 381 employees of the City of Helsinki, Finland. A flow chart of the study is shown in Figure 1. Sufficient data were obtained from 85 participants (9 men, 74 women, and 2 others). The mean age of participants was 42 years (standard deviation [SD] 12.1, range 19–64).

### 2.2 Self‐Reported PA and SB

The HPAQ consisted of questions measuring the total amount of SB and PA (Supporting Information section). The aim of the questionnaire is to identify sedentary and inactive PA profiles among the people living in the City of Helsinki so that health and social care professionals can give the best possible advice on health‐enhancing PA.

The self‐reported PA and SB questionnaire was completed on article. The questionnaire consisted of questions from the HPAQ and questions on PA from validated national [21, 22] and international population surveys [23, 24]. Questions 2.1 and 2.2 assessed SB, and Questions 2.3–3.3 assessed the total amount of PA (Supporting Information section).

### 2.3 Device‐Based Measurement of PA and SB

The reference method for this validation study was a triaxial accelerometer (UKK RM42, UKK Terveyspalvelut Oy, Tampere, Finland). The accelerometer data were analyzed in 6‐s epochs and further smoothed with 1‐min and 6‐min exponential moving averages (EMA). The 6 s time intervals measure even short bouts of PA, whereas the 1 and 6 min EMAs simulate metabolic responses to PA [25]. The accelerometer classifies PA to light (<3 MET), moderate (3.0–5.9 MET), and vigorous (>6 MET) intensities [26]. The UKK RM42 accelerometer has been and is being utilized in numerous Finnish population studies involving several thousand participants, ranging from children to the elderly [10, 22].

## 3 Data Analysis

### 3.1 Data Analysis of PA and SB

The raw acceleration data were submitted without any personal identifiers to the UKK Institute for analysis that was done using validated algorithms developed by the UKK Institute [22, 26]. These analyses provide comprehensive information on the duration of the participant's PA at different intensity levels and the duration of SB (standing, sitting, and lying down) at different times of the day. The analyzed data enable a reliable determination of the participant's PA profile [22]. For the data analysis, participants were divided into two groups according to their PA measured by the accelerometer. The physically inactive group did not meet the weekly PA recommendation assessed by the 6‐min EMA (<150 min weekly MVPA) [6]. The physically active group met the weekly PA recommendation (≥150 min weekly MVPA), respectively. For the fulfillment of the PA recommendation, the time of MVPA was calculated as the sum of moderate PA time and doubled vigorous PA time.

### 3.2 Statistical Analysis

Mean, SD, median, and range are provided as descriptive data. The primary statistical analysis was a binary logistic regression model, where the group (0 = physically inactive, 1 = physically active) was the dependent dichotomous variable, and the explanatory variables derived from the responses to the questionnaires were dichotomous (0/1) or ordinal variables, as appropriate.

The validity of individual questions was assessed using Spearman's correlation coefficients and by examining cross‐tabulations. Physically inactive and active groups provided the comparison method, which was based on the weekly recommended amount of PA (150 min of MVPA used as a cutoff value) and the daily step count, for which the cut‐off between low and sufficient PA was set at 6000 steps per day. The 6000 steps limit represented the lower tertile. For PA questions, MET‐hours were calculated using 2 MET for light PA, 4.5 MET for moderate PA, and 8 MET for vigorous PA [27]. MET‐hours represent the total intensity‐weighted amount of PA during the whole week.

Logistic regression analysis was also used to find the question or pair of questions that would best identify a physically inactive person. We saved the predicted probabilities from each model and used the Mann–Whitney *U* test to examine which question or pairs of questions had the most significant difference between groups. In this examination, the grouping variable was the dichotomous PA recommendation of 21.4 min per day of MVPA in 6‐min EMA (0 = physically inactive, 1 = physically active). The best question or pair of questions created a “traffic light” model: red = physically inactive based on both questions, yellow = physically inactive based on the other question, and green = physically active based on both questions. A similar “traffic light” model was created for SB: red = 10 h or more of SB, yellow = 8–9 h of SB, and green = less than 8 h of SB. The models showed how accurately the questions predict a person's SB and total PA. The size of Spearman's correlation coefficient is interpreted as the following values: 0.00–0.30 negligible correlation, 0.30–0.50 low correlation, 0.50–0.70 moderate correlation, 0.70–0.90 high correlation, and 0.90–1.00 very high correlation. The chi‐square test was used to test the significance of the association between the variables.

IBM SPSS Statistics (IBM SPSS Statistics for Windows, Version 29.0. Armonk, NY: IBM Corp., New York, NY, USA) was used for the statistical analysis.

## 4 Results

Descriptive data for the physically inactive (<150 min weekly MVPA) and active (≥150 min weekly MVPA) are shown in Table 1.

**TABLE 1:** Descriptive background characteristics in the physically inactive and active groups based on the amount of weekly moderate to vigorous physical activity (MVPA) using 6‐min EMAs.

	Total (*n *= 85)	Below 150 min MVPA (*n *= 11)	Above 150 min MVPA (*n *= 74)	*p*
**Age**, years				0.220
Mean (SD)	42.1 (12.1)	46.1 (11.3)	41.5 (12.2)	
**Sex**				0.697
Male, *n* (%)	9 (11)	0 (0)	9 (12)	
Female, *n* (%)	74 (87)	11 (100)	63 (85)	
Other, *n* (%)	2 (2.4)		2 (2.7)	
**BMI**, kg/m^2^				0.002^*^
Mean (SD)	24.8 (4.3)	29.5 (6.2)	24.0 (3.6)	
Median [Min; Max]	23.8 [18.4; 39.9]	29.1 [19.5; 39.9]	23.5 [18.4; 35.1]	
**Sitting** ^a^, min/day				0.030^*^
Mean (SD)	552 (108.2)	621 (126.9)	542 (102.1)	
Median [Min; Max]	543 [349; 843]	594 [392; 832]	534 [349; 843]	
**Standing** ^b^, min/day				0.001^*^
Mean (SD)	128 (53.1)	87 (22.5)	134 (53.7)	
Median [Min; Max]	114 [30; 286]	80.9 [50; 126]	121.3 [30; 286]	
**Steps**				<0.001^*^
Mean (SD)	8130 (3119)	5128 (1710)	8576 (3038)	
Median [Min; Max]	7458 [1666; 20,003]	5229 [1666; 7201]	7761 [3583; 20,003]	
**Light PA** ^c^, min/day				0.995
Mean (SD)	188 (46.8)	185 (67.3)	189 (43.6)	
Median [Min; Max]	186 [79; 296]	193 [79; 292]	182 [119; 296]	
**Moderate PA** ^c^, min/day				<0.001^*^
Mean (SD)	56 (24.5)	29 (10.1)	60 (23.5)	
Median [Min; Max]	52 [9; 142]	30 [9; 47]	60 [22; 142]	
**Vigorous PA** ^c^, min/day				<0.001^*^
Mean (SD)	4.6 (9.4)	0.7 (2.0)	5.2 (10.0)	
Median [Min; Max]	1 [0; 72]	0 [0; 7]	2 [0; 72]	

Abbreviations: BMI, body mass index; EMA, exponential moving average; SD, standard deviation.

^a^ Sitting in 1‐min EMA.

^b^ Standing in 1‐min EMA.

^c^ PA = Physical activity in 1‐min EMA.

* *p* < 0.05.

The correlations of questions assessing SB compared to the accelerometer data are presented in Table S1. The question assessing SB in number of hours had the highest correlation (Spearman's *ρ* = 0.463, *p* < 0.001).

The correlation between the HPAQ questions assessing the total amount of self‐reported PA and accelerometer data is presented in Tables 2 and 3. The highest correlation was for the four‐category question on walking (Spearman's *ρ* = 0.419, *p* < 0.001) of the GPPAQ.

**TABLE 2:** Correlation (Spearman's *ρ*) between self‐reported questions and accelerometer data for total amount of physical activity (PA).

Question^a^	Below vs. above 6000 steps^b^	Below vs. above 150 min MVPA^c^
Question 2.3 on PA (ACSM 2015)	0.218	0.353^**^
Question 2.4 on PA	0.157	0.247
Question 2.5 on PA	0.169	0.219
Question 2.9 on PA	−0.165	−0.186

Abbreviations: EMA, exponential moving average; MVPA, moderate to vigorous physical activities.

^a^ The questions are described in detail in Supporting Information Appendix 1.

^b^ Physical activity groups by step count (dichotomous variable): <6000 steps/day versus ≥6000 steps/day.

^c^ Physical activity groups by MVPA in 6‐min EMAs (dichotomous variable): <150 min of MVPA per week versus ≥150 min of MVPA per week.

* *p* < 0.05.

** *p* < 0.01.

**TABLE 3:** Correlation (Spearman's *ρ*) between self‐reported questions and accelerometer data for total amount of physical activity (PA).

Question^a^	Step count	MVPA in 6‐min EMA
Question 2.8a on PA (walking)	0.404^**^	0.419^**^
Question 2.3b on PA (cycling)	−0.063	−0.027
Question 2.3c on PA (physical exercise)	0.177	0.261^*^
Question 2.3d on PA (strength training)	0.068	0.178
Question 2.3e on PA (housework)	0.135	0.137
Question 2.3f on PA (gardening)	−0.154	−0.180
Question 2.3 on PA (total)	0.226^*^	0.292^**^

Abbreviations: EMA, exponential moving average; MVPA, moderate‐to‐vigorous physical activity.

^a^ The questions are described in detail in Supporting Information Appendix 1.

* *p* < 0.05.

** *p* < 0.01.

The correlation between the questions in the national population surveys and accelerometer data is shown in Table S2. The highest correlation was found for the question of the FinFit2021 survey (Spearman's *ρ* = 0.407, *p* < 0.001).

The traffic light model of the best question on SB is shown in Table S3. The sensitivity of the red light in the model (red vs. yellow + green) was 52%, and specificity was 84%, respectively.

On the basis of the logistic regression models, the combination of two questions was found to have better fit to identify physically inactive subjects than using only one question. Traffic light models of the best pair of questions of total PA are presented in Table 4. The best pair of questions on total PA to identify inactivity were Question 2.3 on PA about 30 min of moderate PA on at least 3 days per week for at least the last 3 months of ACSM [13] and Question 2.4 on PA about average weekly PA from the HPAQ. In Table 4, the sensitivity of the red light in the model (red vs. yellow + green) with the step count was 39%, and the specificity was 81%. The sensitivity of the red light in the model (red vs. yellow + green) with MVPA in 6‐min EMA was 64%, and the specificity was 82%, respectively. For the 18% of sufficiently physically active participants (measured MVPA based on 6‐min EMA) who ended up in the red zone based on the questionnaire, the average daily step count was 8211, with a median of 7885 (min 4474, max 11,432). The questions used in the traffic light models of total PA had an odds ratio of 8.6 (95% confidence interval [CI] = 2.0–36.8, *p* = 0.003) between Question 2.3 and MVPA in 6‐min EMA and an odds ratio of 3.9 (95% CI = 0.4–34.8, *p *= 0.228) between Question 2.4 and MVPA based on 6‐min EMA.

**TABLE 4:** Percentage distribution of the study participants according to the traffic light model of the questions of total physical activity (PA).

		Step count			MVPA in 6‐min EMA		
Questions^a^	PA category	<6000 steps (*n *= 18) (%)	≥6000 steps (*n *= 67) (%)	*p*	MVPA <150 min/week (*n* = 11) (%)	MVPA ≥150 min/week (*n* = 74) (%)	*p*
Question 2.3 on PA	Red	38.9	19.4	0.236	63.6	17.6	0.005
Question 2.4 on PA	Yellow	50.0	62.7		36.4	63.5	
	Green	11.1	17.9		0	18.9	

*Note:* Red = physically inactive based on both questions, yellow = physically inactive based on the other question, and green = physically active based on both questions and a dichotomous variable of accelerometer data. *p* = chi‐square test.

Abbreviations: EMA, exponential moving average; MVPA, moderate‐to‐vigorous physical activity.

^a^ The questions are described in detail in Supporting Information Appendix 1.

## 5 Discussion

### 5.1 Main Results

In this study, we developed a screening tool, the HPAQ, to identify physically inactive people in a social services and health care setting. The tool can be integrated into a digital customer portal of social services and health care, and through this, it can help social and health care professionals promote PA among people at increased risk of non‐communicable diseases. The best question for assessing SB was the question initially planned for the HPAQ that asked about SB in hours. For assessing total PA, the best question was the four‐category question on walking from the General Practice Physical Activity Questionnaire [24]. The questions in the national population survey the FinFit2021 [22] had the best validity to assess MVPA. The best combination of questions to screen physical inactivity was a pair of questions from the American College of Sports Medicine [13] and the HPAQ inquiring about average weekly PA.

The ability of the questionnaires to identify those people who are sufficiently physically active is poor compared to that of accelerometers. Even the combinations of questions did not accurately identify people whose PA was insufficient for their health. A remarkable proportion of the target group was lost if the red group alone was used. If both the red and yellow groups were used, more than 80% of the physically active are included, which makes the screening of those who are physically inactive inaccurate.

The PA recommendations are defined according to the amount of MVPA, which makes the classification of groups according to total PA more reliable [2]. This study analyzes PA during waking hours. The PA recommendations are moving toward promoting optimal distribution of time spent in PA, SB, and sleep behaviors over a 24‐h period, rather than focusing on time spent on each behavior separately [28]. Participants in the physically active group took an average of 8576 steps per day, whereas the inactive group took an average of 5128 steps. For reference, the average number of steps taken by the adult population in the FinFit 2017 study was 7451 steps [10]. A categorization by number of steps can indicate the total amount of PA but does not distinguish the intensity of the activity. Moreover, studies show that commuter cyclists accumulate a large proportion of the PA recommendation by cycling but do not necessarily accumulate steps (depending, however, on the location of the meter and analysis algorithm) [29].

It should be noted that the target group of this study was employed adults of working age, most of whom lived in an urban environment. Among City of Helsinki employees, 17% walk and 31% cycle their commute at least a few times a week. Around one quarter of employees use public transport daily or almost daily to commute to work, including walking or cycling at least 1 km. The work may also include some natural light activity. The questionnaires used in this survey did not separate work and leisure time PA but aimed to count them together as a total. For respondents, this may be more challenging to assess subjectively.

### 5.2 Results in Comparison With Previous Research

In this study, the Spearman correlation between the questions used in the national population surveys and accelerometer data indicated low validity (Spearman's *ρ* 0.29–0.41), which is comparable to previous European studies in terms of validity between the same UKK RM42 accelerometer and PA assessment questionnaires [30, 31]. Charles et al. and Meh et al. studied the validity and reliability of questionnaires to assess PA and SB [30, 31]. The most commonly used PA questionnaires in the EU, the International Physical Activity Questionnaire Short Form (IPAQ‐SF), the Global Physical Activity Questionnaire (GPAQ), and the Physical Activity Questionnaire used in the European Health Interview Survey (EHIS‐PAQ), showed low‐to‐moderate correlations with the accelerometer (Spearman's *ρ* 0.38–0.45) for SB and (Spearman's *ρ* 0.34–0.42) for vigorous PA [31]. In previous studies, the GPAQ questionnaire has been shown to be a suitable and acceptable tool for monitoring PA in population health surveillance systems [32]. The French National Observatory for Physical Activity and Sedentariness Physical Activity Questionnaire (ONAPS‐PAQ) had good reliability (intracluster correlation coefficient = 0.71–0.98; Kappa = 0.61–0.99) and acceptable validity (Spearman's *ρ* = 0.56–0.86) for measuring PA and SB and appeared to give a better estimate of SB than the GPAQ [30].

The questions developed for the HPAQ assessing the total amount of PA had negligible to low correlation with the accelerometer (0.16–0.35), which is less than previous national surveys. The validity of the HPAQ for SB was also low (Spearman's *ρ* 0.39–0.46) but comparable to IPAQ‐SF, GPAQ, and EHIS‐PAQ [31].

Meh et al. reported that adults overestimated MVPA and underestimated SB for more than 2 h [31]. Differences between subjective and device‐based measures of PA may be influenced by the physical fitness of participants [31]. In a study by Heron et al., the GPPAQ was found acceptable for use by general practitioners in all target groups, and the best way to assess patients’ PA was when patients completed the PA questionnaire before coming to see a general practitioner [8].

### 5.3 Practical Implications for Social and Health Professionals

Our aim was to develop and validate a very short tool that could identify patients who do not meet the PA guidelines. When recognized, they could be forwarded to different types of PA promotion. Although even the best performing combination of questions suffered challenges of sensitivity and specificity, we consider its suitability and usability acceptable. Assessing patients’ PA habits already has an activating effect, whereas social and health services have a key role in identifying inactivity and barriers to PA. Naturally, it would be beneficial if more inactive participants could be recognized by this tool, suggesting that further research is needed to refine the existing tools to be more accurate. However, it is well known that even the longer versions of existing PA questionnaires do not recognize the inactive participants significantly better than our short tool [30, 31]. Therefore, given the limited resources available to the social and health care system, it is important to target PA promotion to those who need it most—the inactive people.

### 5.4 Strengths

The strength of the study was the use of a reliable and valid accelerometer‐based reference method [22, 26]. It has been used in many Finnish population–based studies with several thousand participants of different ages [10, 22]. A hip‐worn accelerometer is more likely to reflect waking behaviors than a wrist‐worn device [33]. The study participants did not see the data measured by the accelerometer in real time nor get any feedback. Seeing real‐time data could change activity and habits as an intervention. In addition, the questionnaire included both nationally and internationally used questions.

Another strength was the reasonable sample size, although unbalanced by sex. Taking into account the drop‐out rate of participants, an intended sample was obtained for the analysis. There was variation in the amount of PA in the sample. Participants were recruited for the study regardless of how much PA they do in their daily lives.

Assessing self‐reported PA has its benefits because of cost‐effective data collection on types and domains of PA, suitability for large‐scale population‐level PA assessment, and promotion of PA in health care settings [34]. The strengths of PA questionnaires are the ease of use, low cost, flexibility of data collection, and suitability for large populations and mass screening [14]. Despite the measurement errors and limitations of inaccurate data associated with PA assessment, they are, according to Holmerhorst et al., a feasible and practical way to screen for PA [12].

### 5.5 Limitations

The main limitation pertained to the challenge of getting a sufficient sample size of physically inactive people. In this respect, the sample size was small and therefore a maximum of two questions were used in a logistic regression analysis. In this study, the cut‐off between low and sufficient PA was set at 150 min of MVPA per week according to PA recommendations [6]. The group with the lowest PA had a daily average of 5128 steps. This is higher than the 5000 steps per day that has been used to describe physically inactive people in some other studies [35]. A single week's measurement can inspire a participant to be more active. This could be avoided by measuring for 3–4 weeks [36].

A limitation of the study is that it was conducted in work departments where most employees are women. As a result, 87% of the participants in the study were women. Because most of the participants were women, the validity and generalizability of the tool may not be as well applicable to men. Physical behavior between sexes is known to differ to some extent. On average, women accumulate 8 min more light PA per day than men, whereas men accumulate 3 min more daily MVPA than women in the Finnish working‐aged adult population, according to FinFit 2017 [10]. Background data on weight and height were self‐reported. The mean age of the participants was 42 (range 19–64) years, which does not correspond to the age of the total population visiting social services and health care. The study did not include older people.

There might be inaccuracies in the step count for different types of exercise about how the accelerometer recognizes the movement as a step count. The accelerometer did not specifically measure muscle strengthening and balance activities, which were asked about in the validation questionnaire. Thus, the questions concerning muscle strengthening and balance activities could not be validated using an accelerometer, which is designed to measure endurance‐type PA. On the other hand, this increases the necessity of using a questionnaire to assess PA. Moreover, the accelerometer was not allowed to get wet, and therefore, data such as water running and swimming were not recorded. In addition, the accelerometer recorded cycling as exercise but did not accurately determine its actual intensity.

### 5.6 Conclusions

The highest correlation of the screening questionnaire with the accelerometer data was 0.463 (*p* < 0.001) for assessing SB in number of hours (HPAQ). For total PA, the highest correlation was 0.419 (*p* < 0.001) for the four‐category question on walking from the General Practice Physical Activity Questionnaire [24]. Of the national population surveys, the FinFit2021 survey had the highest correlation, 0.407 (*p* < 0.001), for MVPA.

The best pair of questions on total PA to identify physically inactive participants was the question about 30 min of moderate PA on at least 3 days per week for at least the last 3 months proposed by the American College of Sports Medicine [13] and the question on average weekly PA from the HPAQ. The best pair of questions on total PA identified 64% of all inactive participants in the red zone and 36% in the yellow zone. Using this combination of the PA questions, 64% of all physically inactive participants could be targeted for PA promotion, whereas 18% of all physically active participants could also be targeted for PA promotion. This tool is intended to identify people who are physically inactive in particular. However, even longer versions of existing PA questionnaires do not identify inactive participants significantly better than our tool [30, 31]. However, given the limited resources available to the social and health care system, it is important to target PA promotion to those who need it most—the physically inactive people.

The traffic light model can be used to categorize individuals according to objective data into those who are truly physically inactive and those who are truly sufficiently active and to compare how people estimate their own PA levels in the questionnaire. However, with the present research data, the sensitivity and specificity of the traffic light model were not high enough so that this approach could be used in the clinical setting. The HPAQ has been designed and studied for the working‐age adult population. The suitability of this tool for young adults and older adults needs to be further studied, as PA recommendations for older adults emphasize muscle strength training, balance, and flexibility more than for working‐age adults [6].

The questionnaires developed for screening PA have a poor ability to find people who are physically inactive and at increased risk of non‐communicable diseases. Although screening questionnaires roughly describe the PA level among middle‐aged participants, the clinical setting may require a more accurate method (e.g., accelerometer) to identify and monitor physically inactive people.

## Author Contributions

**Eero Kenttä:** writing – original draft, project administration, data curation, visualization, methodology, funding acquisition, formal analysis, investigation, software, resources, conceptualization, validation. **Harri Sievänen:** writing – review and editing, methodology, validation, conceptualization. **Anastasiya Verho:** writing – review and editing, methodology, conceptualization. **Minna Paajanen:** writing – review and editing, conceptualization, methodology. **Timo Lukkarinen:** writing – review and editing, methodology, conceptualization. **Henri Vähä‐Ypyä:** writing – review and editing, data curation, formal analysis. **Jani Raitanen:** writing – review and editing, formal analysis, software. **Kari Tokola:** writing – review and editing, formal analysis, software. **Tommi Vasankari:** writing – review and editing, supervision, validation, conceptualization, methodology, visualization. **Jari Parkkari:** supervision, validation, conceptualization, methodology, visualization, writing – original draft.

## Ethics Statement

The Human Sciences Ethics Committee at the University of Jyväskylä gave ethical approval for this study. Approval code: 774/13.00.04.00/2023. Approval date: 20 June 2023.

## Consent

All participants signed an informed consent before participation.

## Conflicts of Interest

The authors declare no conflicts of interest.

## Supporting information



Supporting Information

## Data Availability

The data that support the findings of this study are available from the corresponding author upon reasonable request.
