# Supplementary material for: Development and Validation Study of a Screening Questionnaire to Identify People Who Are Physically Inactive
Source: Public Health Chall. 2025 Feb 27;4(1):e70037. doi: 10.1002/puh2.70037 (PMC12039349; doi:10.1002/puh2.70037)
Supplement: Supplementary file 1 — Supporting Information [file PUH2-4-e70037-s001.docx]

**Supplemental material**

**Introduction**

Small health benefits can already be achieved by reducing sedentary behavior (SB) and engaging in even a small amount of moderate to vigorous physical activity (MVPA), even if the full physical activity (PA) recommendation is not achieved [1]. A small amount of movement and PA is better than no movement at all [1], but additional benefits are gained by increasing the amount of time, weekly number of times, and higher intensities of PA [2]. Alongside this, the adult PA recommendation highlights the importance of daily PA, taking breaks from SB, and getting an adequate amount of restorative sleep each day [1]. Increasing PA and reducing SB confers benefits almost to everyone, and it is the least physically active people who benefit most from even a small increase in overall activity levels [2].

**Methods**

***Setting***

Within the social services and health care sector of the City of Helsinki, HUS Helsinki University Hospital, the wellbeing services county of Vantaa and Kerava, and Kauniainen, an electronic social and health care record and its client portal, called Maisa, enables clients to contact a professional and manage their own affairs electronically using a phone app or a computer, anytime and anywhere (Maisa, Oy Apotti Ab, Helsinki, Finland). The client portal Maisa provides a possibility to send a questionnaire to the patient to be answered before the patient contact. A PA assessment or screening questionnaire can be developed for the client portal Maisa.

***Study population***

The participants were invited from library services, maternity and child health clinics, home services for families with children, and health stations and outpatient clinics. The study was presented to the employees face to face at their weekly meetings and by email. The volunteer participants were enrolled in the study via a link in Questback (Questback Oy, Espoo, Finland). The aim was to recruit 100 participants aged 18–64 years to achieve a sample size of *n* ≥ 60 (estimated drop out 30%–40%).

The inclusion criteria were age between 18­ and 64 years, and that the participants had no symptoms preventing PA according to the ACSM’s recommendations [3]. A participant was excluded if they did not want to use the accelerometer. The data of a participant were excluded from the analysis if their accelerometer data were insufficient (at least 10 hr/day for 4 days) or if the participant considered the measurement week as being different from their normal week. This latter issue was inquired about in the study questionnaire.

This study was conducted between September and November 2023. Participants wore the accelerometer for seven days, after which they answered the self-report questionnaire. The questionnaire included a personal survey ID and a summary of the survey information in an envelope. The participants received both oral and written instructions on how to use the accelerometer, and immediately started using it. Those who did not attend the information session received an accelerometer, a questionnaire, and written instructions by internal mail.

***Self-reported physical activity and sedentary behavior***

The questionnaire was designed using research findings and, where applicable, questions from national survey questionnaires. The questionnaire also included questions and answer options developed or applied by the PA and medical specialists themselves.

***Device-based measurement of physical activity and sedentary behavior***

The accelerometer continuously measures the raw acceleration data caused by all movements at a sampling frequency of 100 times per second. The participants wore the accelerometer on their right hip in a special belt case during the measurement period and throughout their waking hours for seven days. A valid wear time for an accelerometer is at least four days and 10–19 hours/day. Overall, this threshold can be regarded as adequately valid for representing the total amount of SB and PA during waking hours [4].

**Discussion**

***Strengths***

The survey was conducted in autumn, which is working time in Finland, and the participants were not on vacation. Therefore, the time of measurement apparently reflected the participants’ everyday life. During the period of data collection in September–November 2023 the average temperatures in Helsinki were between 0.8 and 15.8 °C and there was no snow or ice to prevent PA, which increased the reliability of the survey. The COVID-19 pandemic did not limit PA or participation in organized activities or sports facilities at the time of the study.

**Supplementary table 1**. Correlation (Spearman’s ρ) between self-reported questions and accelerometer data for sedentary behavior (SB)

| Question^1^ | SB in 6-s epoch | SB in 1-min EMA |
| --- | --- | --- |
| Question 2.1 on SB  Question 2.2 on SB | 0.389**  0.463** | 0.385**  0.453** |
| ^1)^ The questions are described in detail in Supplemental material * *p* < 0.05  ** *p* < 0.01  SB = Sedentary behavior, EMA = Exponential moving average | | |

**Supplementary table 2**. Correlation (Spearman’s ρ) between self-reported questions in MET-hours and accelerometer data for total amount of physical activity (PA)

| Question^1^ | Step count | MVPA in 6-min EMA |
| --- | --- | --- |
| Question 3.1 National FinSote Survey 2020  Question 3.2 FinFit 2021  Question 3.3 Capital Region Wellbeing Survey 2021 | 0.383**  0.407**  0.289** | 0.393**  0.389**  0.323** |
| ^1)^ The questions are described in detail in Supplemental material  * *p* < 0.05  ** *p* < 0.01  MVPA = Moderate to vigorous physical activity, EMA = Exponential moving averages | | |

**Supplementary table 3**. Percentage distribution of the study participants according to the traffic light model of the question on sedentary behavior (SB): Red = 10 hr or more of SB, Yellow = 8–9 hr of SB, and Green = less than 8 hr of SB and a dichotomous variable of accelerometer data

| **Question^1^** | **SB category** | **SB in 6-min EMA** | |  |
| --- | --- | --- | --- | --- |
|  |  | **≥10 hr of SB (*n* = 21)** | **<10 hr of SB (*n* = 64)** | ***p*** |
| Question 2.2 on SB | Red | 52.4% | 15.6% | 0.005 |
|  | Yellow | 28.6% | 42.2% |  |
|  | Green | 19.0% | 42.2% |  |

^1)^ The question is described in detail in Supplemental material

*p* = Chi-square test

SB = Sedentary behavior, EMA = Exponential moving average

**Appendix 1. Translated Questions of the Questionnaire in the Study.**

**Question 2.1 on SB** (Helsinki Physical Activity Questionnaire)

How many hours do you sit or have sedentary activities on a typical day?
*Take into account any waking time spent sitting or lying down, such as eating, watching TV, reading, studying, working, using a smart device and travelling in transport.*

a) Less than 4 hours per day
b) More than 4 hours per day but less than 7 hours per day
c) More than 7 hours per day but less than 10 hours per day
d) More than 10 hours per day.

**Question 2.2 on SB** (Helsinki Physical Activity Questionnaire)

How many hours do you sit or have sedentary activities on a typical day?
*Take into account any waking time spent sitting or lying down, e.g., eating, watching TV, reading, studying, working, using a smart device and travelling in transport.*

a) 0 hour b) 1 hour c) 2 hours d) 3 hours e) 4 hours f) 5 hours
g) 6 hours h) 7 hours i) 8 hours j) 9 hours k) 10 hours l) 11 hours
m) 12 hours n) 13 hours o) 14 hours p) Over 15 hours per day

**Question 2.3 on PA** (ACSM 2015)

The American College of Sports Medicine (Riebe et al. 2015):

Exercise participation, performing planned, structured physical activity at least 30 minutes at moderate intensity on at least 3 days per week for at least the last 3 months.
a) Yes
b) No

**Question 2.4 on PA** (Helsinki Physical Activity Questionnaire)

What kind of physical activity do you do each week on average?
*Physical activity in this context refers to all physical activity in everyday life and leisure time. Think about your day, including commuting to and from work, housework, gardening, physical activity at home, walking the dog, nature and exercise in your leisure time. If necessary, answer more than one option.*

a) I do not regularly do physical activity in my daily life or in leisure time (I mainly use public transport, my work is not physically demanding, I do not have any leisure activities)
b) I have regular daily physical activities (I am active in my daily life, I walk or cycle at least part of the way to work and to the shop, take the stairs, garden, walk the dog).
c) I play recreational sports in my leisure time (e.g., outdoor activities, gym or strength training, jogging, cycling, swimming, gymnastics, ball games, dancing, hiking in nature).

**Question 2.5 on PA** (Helsinki Physical Activity Questionnaire)

How much physical activity and exercise do you do on average during your work and leisure time?
*If activity varies widely, for example between seasons, try to estimate the average. The question concerns the past year. If necessary, answer more than one option.*

a) In general, I don’t do much physical activity, and in leisure time I mainly read, watch TV, work on the computer, do tasks that don’t physically stress me, or otherwise spend my time sedentary or resting.

b) I do some light exercise such as cycling or walking to work, outdoor activities, gardening, walking in nature or other similar activities.

c) I regularly do at least 2 ½ hours of moderate physical activity per week, such as heavy gardening, brisk walking or cycling, running, swimming, skiing, tennis, badminton, and similar activities.

d) I regularly do at least 1 hour and 15 minutes of heavy or competitive training per week, such as running, orienteering, skiing, swimming, football, ice hockey and similar sports.

**Question 2.8 on PA**

Adapted General Practice Physical Activity Questionnaire (according to Department of Health and Social Care, United Kingdom 2013)

During the last week, how many hours did you spend on each of the following activities? Please answer whether you are employed or not.
*Please mark one box only on each row.*

|  | None | Some but less than 1 hour | 1 hour but less than 3 hours | 3 hours or more |
| --- | --- | --- | --- | --- |
| a) Walking, including walking to work, shopping, leisure, etc. |  |  |  |  |
| b) Cycling, such as commuting to work and leisure, but not competitive cycling. |  |  |  |  |
| c) Other physical exercise such as swimming, jogging, gymnastics, football, tennis, gym training, skiing, horse riding, etc. |  |  |  |  |
| d) Strength training (e.g., circuit training, Pilates or gym training, with at least 8–12 reps of exercises on different muscle groups) |  |  |  |  |
| e) Housework inside including childcare |  |  |  |  |
| f) Housework outdoors such as gardening, lawn mowing and shoveling snow |  |  |  |  |

**Question 2.9 on PA** (Helsinki Physical Activity Questionnaire)

Do you feel your physical activity is sufficient to support your health?
a) Yes
b) No

**Question 3.1 National FinSote Survey 2020** (Parikka et al. 2020)

How much physical activity do you do each week for work, commuting and leisure?
*Think about the last 12 months. Consider all regular weekly physical activity. Answer on more than one line if necessary.*

a) Hardly any regular weekly physical activity

b) Slow and gentle movement (= no sweating or accelerated breathing, e.g., slow walking)

__ days per week, for a total of __ hours and __ minutes per week

c) Brisk, moderate physical activity (= some sweating and/or accelerated breathing, e.g., brisk walking)

__ days per week, for a total of __ hours and __ minutes per week

d) Strenuous and vigorous exercise (= heavy sweating and/or accelerated breathing, e.g., jogging or running)

__ days per week, for a total of __ hours and __ minutes per week

**Question 3.2 FinFit 2021** (Husu et al. 2022)

Think about the last year (12 months). Please circle all the options from 2 to 6 that correspond to your situation, and mark on the lines how much physical activity you do (days per week, hours and minutes in total per week). Include any physical activity that you do regularly on a weekly basis. If you do hardly any regular weekly physical activity at all, circle option 1.

1. Hardly any regular physical activity every week

2. Slow and gentle endurance activities (= no sweating or accelerated breathing, e.g., slow walking)

__ days per week, for a total of __ hours and __ minutes per week

3. Brisk, moderate endurance activities (= some sweating and/or accelerated breathing, e.g., brisk walking)

__ days per week, for a total of __ hours and __ minutes per week

4. Strenuous and vigorous endurance exercise (= heavy sweating and/or accelerated breathing, e.g., jogging or running)

__ days per week, for a total of __ hours and __ minutes per week

5. Muscle training (e.g., a circuit or gym workout, with at least 8–12 movements for different muscle groups) __ days per week, for a total of __ hours and __ minutes per week

**Question 3.3 Capital Region Wellbeing Survey 2021** (Ahlgren-Leinvuo et al. 2022)

1. How much physical activity do you do each week for work, commuting and leisure?

Think about the last 12 months. Consider all regular weekly physical activity. Select all suitable options.

a) Hardly any regular weekly physical activity

b) Slow and gentle movement (= no sweating or accelerated breathing, e.g., slow walking)

__ days per week, for a total of __ hours and __ minutes per week

c) Brisk, moderate physical activity (= some sweating and/or accelerated breathing, e.g., brisk walking)

__ days per week, for a total of __ hours and __ minutes per week

d) Strenuous and vigorous exercise (= heavy sweating and/or accelerated breathing, e.g., jogging or running)

__ days per week, for a total of __ hours and __ minutes per week

2. How many days per week do you usually do physical activity to maintain or develop your muscle strength and/or mobility training? For example, gym, exercise at home, group exercise, ball and racket games or physically demanding activities. If not at all, answer 0.

__ times.

**References:**

1. Physical activity guidelines advisory committee., “Physical activity guidelines advisory committee scientific report,” (Washington, DC: U.S. Department of Health and Human Services, 2018), accessed 2023, <https://odphp.health.gov/our-work/nutrition-physical-activity/physical-activity-guidelines/current-guidelines/scientific-report>.

2. K. Piercy, R. Troiano, R. Ballard, S. Carlson, J. Fulton, D. Galuska, S. George, and R. Olson, “The Physical Activity Guidelines for Americans,” JAMA 320 no. 19 (2018): 2020–2028, <https://doi.org/10.1001/jama.2018.14854>.

3. D. Riebe, B. Franklin, P. Thompson, C. Garber, G. Whitfield, M. Magal, and L. Pescatello, “Updating ACSM’s Recommendations for Exercise Preparticipation Health Screening,” Medicine & Science in Sports & Exercise 47 no. 11 (2015): 2473–2479, <https://doi.org/10.1249/MSS.0000000000000664>.

4. Husu, K. Tokola, H. Vähä-Ypyä, H. Sievänen, J. Suni, O. Heinonen, J. Heiskanen, K. Kaikkonen, K. Savonen, S. Kokko, and T. Vasankari, ”Physical Activity, Sedentary Behavior, and Time in Bed Among Finnish Adults Measured 24/7 by Triaxial Accelerometry,” Journal for the Measurement of Physical Behaviour 4 no. 2 (2021): 163–173, <https://doi.org/10.1123/jmpb.2020-0056>.

5. World Health Organization, “Global physical activity questionnaire (GPAQ),” (2021), accessed October 6, 2023, <https://www.who.int/teams/noncommunicable-diseases/surveillance/systems-tools/physical-activity-surveillance>.

6. Department of Health and Social Care, United Kingdom, “General practice physical activity questionnaire (GPPAQ),” (2013), accessed October 6, 2023, <https://www.gov.uk/government/publications/general-practice-physical-activity-questionnaire-gppaq>.

7. S. Parikka, T. Koskela, J. Ikonen, H. Kilpeläinen, L. Hedman, S. Koskinen, and A. Lounamaa, ”National FinSote Survey,” (2020), accessed October 6, 2023, <https://thl.fi/finsote>.

8. P. Husu, H. Vähä-Ypyä, K. Tokola, H. Sievänen, A. Mänttäri, S. Kokko, K. Kaikkonen, K. Savonen, and T. Vasankari, ”Measurement of Physical Fitness and 24/7 Physical Activity, Standing, Sedentary Behavior, and Time in Bed in Working-Age Finns: Study Protocol for FINFIT 2021,” Methods and Protocols 5 no. 1 (2022): 7, <https://doi.org/10.3390/mps5010007>.

9. H. Ahlgren-Leinvuo, J. Erjansola, M. Joensuu, N. Mäki, M. Mänty, and A-P. Sihvonen, “Pääkaupunkiseudun asukkaiden hyvinvointi ja terveys – Pääkaupunkiseudun hyvinvointikyselyn 2021 tuloksia,” (2022), accessed October 6, 2023, <https://kaupunkitieto.hel.fi/fi/paakaupunkiseudun-asukkaiden-hyvinvointi-ja-terveys-paakaupunkiseudun-hyvinvointikyselyn-2021>.
